# Notes on Characteristics

**Published:** 1887-12

**Authors:** C. Carleton Smith, Edmund J. Lee

**Affiliations:** Philadelphia; Philadelphia


### CLINICAL NOTES ON CHARACTERISTICS.

The purpose of these "Notes" is to give in a condensed form the positive characteristics of each remedy as far as known. Each drug has its common, its peculiar, and its characteristic symptoms. It is these latter we are considering. The articles on each drug will be as brief and as condensed as possible, giving only such symptoms as singly or collectively call for the one remedy. Comparisons are given wherever useful to differentiate between closely allied symptoms. The comparisons are few, and restricted to cases where symptoms are characteristic and resemble one another closely. Corrections and additions will be gladly received and published if sufficiently useful.

C. CARLETON SMITH, M. D., } PHILADELPHIA.

EDMUND J. LEE, M. D., }

ABIES CANADENSIS: The action of this remedy, as far as known, seems to be chiefly upon the digestive organs, producing a gnawing, hungry sensation at epigastrium (Grat., Phos., Sep.), with cravings for meat, pickles, and coarse food (resembling Alumina).

There is also a chilliness as if the blood were turned into ice-water (Rhus); a coldness between the shoulders as from cold water. (As from a cold hand, Sepia; as from a lump of ice, Lachn.; all suffering parts feel cold, Sil.)

ABIES NIGRA: Patient is low-spirited, melancholy; has an habitual and severe pain in stomach after eating. A very distressing sensation as of a "hard-boiled egg in stomach." A continued feeling as of constriction just above the pit of the stomach, as if everything were knotted up, or as if a hard lump of undigested food remained there. (In œsophagus: Kali bich., Lactic-acid, Zinc.; above cardiac orifice: Ignatia; foreign body sticking in cardiac orifice: Nat-m.) Wakeful at night with hunger (hungry at night: Bry., Canth., Lyc., Phos., Psor., Tellur.; hunger prevents falling sleep: *Marum;* dreams he is hungry, which awakens him: Arg-n.; immoderate hunger, especially at night, weak, faints if not satisfied: Phos.).

In chronic cases of intermittent fever, with stomach-ache, Abies nigra may be indicated. (Calc., chill commences in pit of stomach, with cold, agonizing weight.) The symptom, pressure or load in stomach after eating, which is prominent under this remedy, is found under many others. Kali bich. has it immediately after eating; Calc., after a moderate supper; Bry., Merc., Nux vom., Puls., pressure as from a stone; Sepia has pain after simplest kind of food and also pressure as from a stone.

ABROTANUM (Artemisia abrotanum): Cross, irritable children, with evident signs of chlorosis or emaciation; appetite is ravenous, yet child loses flesh (Iod., Nat-m.; Calc-ph., child wants to nurse all the time, loses flesh, can't stand; Cina, child is cross, weeps, has great hunger, pale, sickly face, but not so emaciated).

Food passes undigested; face looks wrinkled as if old. (Creos., Sulph.; Op. the suckling of a few weeks did not grow, but looked like an old man.) Weak, cannot hold head up. (Also Æth., Ant-t., Arn., Bapt., Lyc., Mang., Puls., Oleand., Sil., Tabac., Zinc.) Boys with nosebleeding, or hydrocele. Blood and moisture oozing from navel of new-born children.

Rheumatic pains, lame and sore, worse morning on waking (Bry.). Hemorrhoids, burning when touched or during stool. (From touch: Phos., *Sulph.;* during stool: Coc-c.; relieved by stool: *Caust.;* worse after stool: Berb., Nitr-ac.) Hemorrhoids become worse as rheumatism abates. (Lumbago alternating with headache, or with piles: Aloe.)

In children, after influenza, great weakness and prostration and a kind of hectic fever (compare Samb. and Senega). The pains in stomach are worse at night; rheumatic pains are worse on moving, and morning on waking.

ACALYPHA INDICA: Patient is despondent, gloomy; has tuberculosis, with a violent, dry cough, followed by expectoration, or hemorrhages, of pure blood (Cactus). Blood is pure in morning, dark and clotted in evening. Cough worse at night.

ACETIC ACID: Aconite has probably been frequently administered in cases where Acetic acid was the proper remedy. The Acetic acid patient is pale, emaciated; seldom or never fat, and of a waxen color; its mental state is apt to be one of anxiety—worry about sickness or affairs—and irritability. The Aconite symptoms appear suddenly and are violent (the reverse of Acetic acid); the patient is not pale or emaciated; rather red, florid, and plethoric; is anxious, that is, fearful, timid; afraid he will die, *afraid of everything,* is restless. Both Aconite and Acetic acid have thirst, the latter very markedly, and here it differs from Apis. Apis patient is also pale, waxen, but is more apt to be puffed in appearance than emaciated. In dropsies the emaciation and thirst of Acetic acid differ from the puffed, swollen face and the lack of thirst of Apis.

Acetic acid has a nervous headache from abuse of narcotics; from abuse of alcohol, coffee, opium, or tobacco (Asar., Calad.). Pain across root of tongue, impeding speech and motion of jaw. (Sulphur: dull pain in root of tongue, worse evening, obstructing speech.)

Thirst intense, insatiable; shrieks for water at night, notwithstanding has drunk copiously. In dropsies, diabetes insipidus, chronic diarrhœa, greatest thirst; in fevers there is no thirst.

In croup there is a hissing respiration, with rattling in throat, worse at each inhalation (Aconite, at each exhalation). A white film low down in fauces.

If thirsty, water is swallowed with difficulty. Hydrophobia patient springs out of bed and crawls on the floor howling with pain. (Arsenic, rolls about on floor with despair of life from pain in abdomen.)

Patient cannot sleep lying on back, feels as if abdomen were sinking in, causing difficult breathing; rests easier lying on abdomen. Sleep is poor; is disturbed without known cause.

Hemorrhages from nose, lungs, bowels, hemorrhoids, uterus, etc., in *debilitated, pale, thin persons with a waxy appearance.* Is useful after stings, bites, etc.

Aconitum napellus: The key-note of Aconite is *fear;* the patient is never cheerful and contented. Great fear and anxiety, with nervous excitability. Patient's life is rendered miserable by this all-pervading fear; he is afraid to go out doors, to cross a street, or be in a crowd. (These last will be found in chronic cases.) A great anxiety and fear about recovery (also Ars., Bry., Calc., Lach., Plat., etc.); changing mood; cheerful at one time; weeps at another (Ign.). Patient will say he cannot bear the pains any longer; *will die* if not soon relieved. (Allium cepa, patient fears the pains *will become* intolerable.) The patient, when in pain, or during labor, believes she will die and predicts the time. (Agnus patient believes she will die soon, after awhile.) A pregnant woman has been frightened, and the fear remains (also Opium). In labor there is anxiety, distress, and restlessness with every pain; hot and feverish. Children fear to go to bed. Patients fear loss of reason (Calc.).

(Complaints from fright, Hering says: "Soon Opium, later Aconite; fear remaining: Bell., Merc., Nat-m., *Verat.;* fear: Op., Samb.; fear and anger: Acon.; fear and anxiety: Lyc.")

(Many remedies have this fear of death, but none of them has the peculiar Aconite line of symptoms accompanying this fear. It would be impossible to differentiate between these remedies in these *Notes;* however, it will be found that each remedy having this fear of death has its own peculiar symptoms.

Fear of death during hot stage is Calc., Cocc., Ipec., Mosch., *Nit-ac.*, Ruta; during sweat is Nitr.; during menses: Acon., Plat., and Verat.; of sudden death: Ars.

There is another class of remedies, which have rather a desire for death than a fear of it. It may be well to note one or two of these remedies here in contrast with Aconite.

The Aconite patient *fears* death but believes will—that is *must*—die, as the sufferings are *so severe*. *Predicts* the day and bids friends good-bye.

The Aurum patient is melancholic, looks on the dark side of everything, with *constant* thoughts of suicide. Is weary of life and longs to be rid of its troubles. The labor pains make her desperate, would like to jump out of window, etc.

The Argentum nit. patient is also melancholic, or hypochondriac; is full of fanciful notions, such as thinking he cannot walk past a given point without falling, or houses will close upon and crush him, or believes he will suddenly fall down and die, etc. Like Aconite, he will settle upon the time of his death, but this is from melancholy, not on account of the pains.

The Psorinum patient is also anxious and oppressed; is hopeless of recovery. During convalescence he will be hopeless, considering himself too sick to recover. Returning to Aconite, we note :)

The headache is of the congestive type, red, hot face. Burning headache, as if brain were moved by boiling water. Headache is worse from motion, light, noise, and when rising; better lying down in dark, and from cold. Fainting on rising from a recumbent position (Op.); the red face becomes pale (Verat.).

EYES: Ophthalmia, very painful, especially when caused by foreign bodies. (Calc., Sulph.; Sil., and Symph., when from blows, splinters, etc.)

EARS: Aversion to noises; they are intolerable; they trouble and worry the patient. Music seems to go through every limb; makes her sad. (Sadness, Lyc., Nat.-s.; also causes great excitement: Tarent.)

NOSE: Sense of smell very acute, especially for disagreeable odors. Epistaxis, especially in young, plethoric persons, who bleed profusely (Elaps; profuse but soon ceasing: Cact.), and when patient is afraid of bleeding to death. Epistaxis with copious or suppressed menses in plethoric women (in amenorrhœa, Bry., Cact., Ham., Puls.). Dry coryza, with headache, fever, thirst, caused by exposure to dry, cold weather. Checked coryza, with headache; better in open air; worse from talking. Nose dry, stopped up so cannot breathe through it, must breathe through open mouth (also Arum-t., Lyc., Nat-ars., Samb.; stopped up, yet discharges fluids: Arum-tri., Nitr-ac.; stoppage alternating with fluent discharge: Ant-t., Mag-c., Sil.). Fluent coryza with dropping of clear water (Agar.), which is hot.

FACE: Red, hot, and puffed. (Bell. face is purplish; darker red than Aconite.) Red and pale alternately; one cheek red, other pale (Arn., Cham., Ign., Ziz.; Cham. red and hot during pain).

When rising up from recumbent posture red face becomes pale (Verat.). Tingling in cheeks; so red it tingles.

Perspiration on side of face he lies on. (Sil., side does not lie on.)

MOUTH: Lips dry, black, peeling off; burning and numbness of lips and mouth.

Constantly moving lower jaw as if chewing. (Mosch., Phos.; Bry. in sleep; Ars., Bell. Stram., convulsive.)

Tongue coated white. (Ars., Bism.; Ant-cr. white and thick coat.)

Toothache from cold, *throbbing* in one side of face; intense redness of cheek; congestion to head and great restlessness. Teeth sensitive to the air. (Berb., Nat-m., Oxal-ac.; to cold, *Calc.*, Nat-m.)

APPETITE, TASTE, ETC.: Taste bitter; everything tastes bitter *except water* (Stann.).

Burning thirst; drinks little and often (Ars.). Desires wine, brandy, *beer*, and bitter drinks.

THROAT: Burning, dryness, redness of the parts; burning along dorsum of tongue through the œsophagus to stomach. (Ars., Iris-v., Kali bi., Nitr-ac., Sul-ac.)

Acute inflammation of throat, with high fever, dark redness of parts, burning and stinging (Apis, Bell.).

VOMITING: Vomiting with anxiety, heat, nausea, and thirst; with profuse sweat and increased micturition. Patient will drink, vomit, and declare he will die. The vomit pours out profusely as from a pump. Vomiting of what has been drunk, followed by thirst. The vomit may consist of mucus, bile, or blood, etc.; vomiting and purging of green water in cholera.

STOMACH: Sudden excruciating pain, with gagging, retching, vomiting of blood and retching; cold sweat on forehead. (Compare Verat. and Camph.).

When breathing pit of stomach is drawn back to spine (in croup); (when vomiting, Verat.)

ABDOMEN: Colic forces patient to bend double, yet not relieved in any position. Sharp, shooting pains through abdomen; it is *very* sensitive to touch (Bell., Lach., Puls.); hot, tense, tympanitic.

ANUS AND STOOL: Shooting pains and constant pressure in anus.

Stool white with dark urine; frequent small stools with tenesmus and sweat. Dysenteric stool during hot days and cold nights. Green, watery diarrhœa; stools like chopped spinach (Arg-n.). Diarrhœa from getting wet, with slimy, bloody stools, violent pain, restlessness, etc.

URINARY: Inflammatory symptoms of bladder and kidneys, with constant urging, water passing in drops, bloody, with burning. Urine red or dark. Dysuria during pregnancy. Retention in babies for first few days after birth; also in children from cold, with crying and restlessness; enuresis with thirst; diuresis with headache and profuse sweat. Children finger the genitals (Merc., Zinc.).

SEXUAL ORGANS, MALE: Testicles pain as if bruised; feel hard, as if surcharged with semen. Gonorrhœa, with the urinary and mental symptoms of Aconite.

FEMALE: Menses profuse with plethoric women; late, oftener scanty than profuse in other women. The marked action of Aconite upon menstruation is to restore menses suppressed (especially with plethoric women) by cold, by fright, or fear. The pains in uterine region are of sharp, shooting character. It cures sudden prolapsus uteri, with inflammation, vomit, cold sweat, or hot, dry skin.

In pregnancy, Aconite is often indicated for fever, restlessness, and anxiety. In labor, where the pains are very distressing to the patient, the vagina hot and dry. Abortion threatens after a fright (also Opium).

The labor pains are violent, rapid, and distressing; face red; patient shrieking; fœtus seems to be immovable. These symptoms cause great anxiety and fear of recovery on part of patient. (Cham. patient is cross, irritable, won't give a civil answer. Coffea, like Aconite, has fear of death; bright-red face in labor and other troubles. The Coffea patient is irritable, restless, cries and laments, and the pains are unbearable. Aconite has *foreboding*, prediction of death or non-recovery; Coffea has *fear* of death, no predicting of its occurrence or time.) Suppression of the lochia from cold or fright; patient has red face, heat, restlessness, anxiety, etc.

LARYNX AND TRACHEA: Laryngitis with the inflammatory and mental symptoms.

Larynx sensitive to touch, and inspired air, as if denuded. Complaints from straining the voice (Arg., Arn., Arum tri).

CROUP: Patient awakes in first sleep; dry, short cough, but not much wheezing or sawing respiration; cough and loud breathing during expiration (during inspiration: Acetic acid and Spongia, which has sawing, wheezing between coughs); every expiration ends in a hoarse, hacking cough. Patient is in agony, is hot, restless, and feverish, caused by exposure to dry, cold air.

Aconite has dry and clear, or hoarse and hollow coughs; the cough is worse after eating or drinking at night, lying on side; better lying down (Euphr., Mang.) on back; generally caused by exposure to dry, cold air. Patient is nervous, feverish, restless, etc., wants to cough but cannot, in croup. Also coughs after midnight, the more he tries to suppress it the more severe it becomes. (Ign., the irritation to cough ceases if cough be suppressed; Marum verum, the irritation cannot be suppressed but becomes worse as coughing continues.) A continuous, short, hacking cough, with agonized *tossing* about, calls for Aconite. (When patient is *quiet* study Squilla.) Child grasps at throat with each cough. (Grasps at throat with each cough is Acon. or All-c.; in croup: Iod., Lach.; in delirium: Stram.)

RESPIRATION: Labored, anxious, short; asthma from congestion, after scarlatina, with heart troubles, fear, anxiety, restlessness accompanying.

Agony, patient suddenly sits straight up in bed can hardly breathe; pulse threadlike; sweat, anxiety, fear; constant, short, dry cough, with feeling of suffocation, which increases with every inspiration. (Must sit up to breathe: Ant-t., Ars., Bry., Ferr., Hep., Kali-b., Lach., Lact., Spong.) (The Lachesis patient awakes suffocating, fears is dying; grasps throat; especially indicated when aggravation seems to come on during sleep. The Spongia patient, with heart disease, is suddenly awakened after midnight with violent pain, gasping respiration, suffocating feeling, great alarm and anxiety. The Arg-n. patient is in such agony from dyspnœa that he thinks of killing himself to escape it.)

CHEST: Tightness, oppression; must breathe deeply, feels as if chest would not expand (Bry.). Pains are sharp, shooting stitches, transient, now here, now there; are felt most when breathing, coughing (Bry.).

In pleurisy patient cannot lie on right side, only on back. (Bell. lies on painless side; Bry. on painful.) Heart, palpitation, worse lying, with anxiety, restlessness.

Aconite is frequently called for in diseases of the heart, when its mental state is present and there is numbness in left arm and tingling in fingers. (Also Ailan.) Fainting with tingling; palpitation with tingling.

BACK, EXTREMITIES: Numbness in small of back, extending to limbs. Tingling in back.

Arms hang powerless, as if paralyzed by blows. Numbness of left arm, can hardly move fingers. (See Agar.)

Shooting, tearing, erratic pains.

Creeping or tingling in fingers, even while writing. Hands hot, feet cold.

Shooting, tearing pains in legs, knees, ankles, toes, etc. Legs feel almost powerless, numbness after sitting. Rheumatism of hip or knee joint, with heat, throbbing, shooting pains.

Sensation as if drops of cold water trickled down thighs.

Coldness of feet up to ankles, or only of toes, with perspiration of toes and soles of feet.

SLEEP: Nervous restlessness; tosses about; worse from the heat of bed.

Awakened by asthma, nightmare, dry cough, with starts.

FEVER, ETC.: Chill, anxious, ascends from feet to chest; on slightest movement, or being uncovered.

Heat, with agonized tossing about; heat with thirst, full and frequent pulse, impatient, anxious tossing about; thirst, profuse sweat; worse while sweating, better afterward. Cold sweat.

Bad effects from suppressed sweat.

(Aconite fever and thirst, etc., come on in evening and last all night; with Bell. they begin about three P. M. and last till three A. M.)

SKIN: Tingling over surface.

Red, shining, hot swellings. Fine prickings, as from needles here and there (Lobel-inf.). Scarlet rash with high fever.

PECULIARITIES: Pains are intolerable, worse at night (Aur., Merc.). Tingling and numbness in affected parts. Pains of a shooting, cutting character; erratic.

(The Aconite pains are intolerable; the patient says they are so bad he *will* die. The Arsenic patient suffers so from pain that he is determined to kill himself; is also anxious, depressed; has fear, restlessness, but generally weakness and prostration. The Arnica pains are intolerable, drive him crazy; worse from motion or noise; change quickly from part to part; patient scratches at bed or wall, apparently for relief.)

Painful sensitiveness to contact; does not want to be touched (Arn., Iod.).

Inflammatory and congestive symptoms with the mental and nervous concomitants.

When at rest patient is better, but during night in bed pains are intolerable.

Acts upon upper right, lower left side. Remission during day and before midnight. Vegetable acids suspend the action of Aconite; abuse of Aconite calls for Sulphur. When Aconite seems no longer to act then study Bell., Bry., or Calc.

CONDITIONS, aggravation: evening, and after midnight; assuming erect position; when rising from bed; lying on left side; in warm room (of congestive and catarrhal troubles while rheumatic are ameliorated); bad effects from suppressed perspiration, from fright, fear or violent anger, and especially after exposure to dry, cold air.

AMELIORATION: During day; in open air (nervous symptoms); when sitting still (rheumatism); sitting erect; by closing eyes; lying on right side; on expiration; after perspiring; on letting diseased limb hang down; moistening the part; washing with cold water; in wet weather; children by being rocked or carried; anxiety somewhat relieved by cold drinks.

[TO BE CONTINUED.]

